# Education as a Predictor Factor for Knowledge of COVID-19 in Portugal

**DOI:** 10.3389/fpubh.2021.680726

**Published:** 2021-09-29

**Authors:** Joana Gomes da Silva, Carla Sofia Silva, Bárbara Alexandre, Pedro Morgado

**Affiliations:** ^1^Unidade de Cuidados de Saúde Personalizados Mirandela II, Unidade Local de Saúde Do Nordeste, Mirandela, Portugal; ^2^Alumni Department of Mathematics, University of Minho, Braga, Portugal; ^3^Life and Health Sciences Research Institute (ICVS), School of Medicine, University of Minho, Braga, Portugal; ^4^ICVS/3B’s, PT Government Associate Laboratory, Braga, Portugal

**Keywords:** health literacy, COVID-19, education, community, communication, medical sciences

## Abstract

**Introduction/Objective:** Pandemic of COVID-19 is a major public health problem. At the time of development of this study, no specific medication/vaccine for this disease was approved. Therefore, preventive measures were the main key to control this pandemic. Health literacy (HL) is the ability to obtain, understand and use the information to make free and informed decisions about the health of an individual and to promote community empowerment. Thus, the HL of COVID-19 is important for community empowerment and the adoption of preventive measures. This article aims to understand possible predictors of HL of COVID-19, functional domain.

**Material and Methods:** A cross-sectional study was designed, applying the Questionnaire of COVID-19 (previously designed and submitted to a preliminary pilot testing) through an online platform from April 23 to June 23, 2020. An Index of Health Knowledge of COVID-19 Questionnaire (IHK-COV19) was constructed. Associations between independent variables (“Gender,” “Age,” “Education,” and “Risk Factor” for COVID-19 codified by ICPC-2) and HL were assessed using multivariate analyses (mixed effects models). The level of significance was set at *p* < 0.05.

**Results:** Our sample includes 864 subjects (median age, 44.33 years), mostly women (*n* = 619; 71.76%), undergraduate (*n* = 392; 45.37%) and with at least one risk factor for COVID-19 (*n* = 266; 30.79%). Univariate and multivariate analyses demonstrated “Age” as a negative predictor of IHK-COV19 and “Education” and “Risk Factor” as positive predictors of IHK-COV19.

**Conclusions:** Health knowledge regarding COVID-19 is associated with the level of education. Future interventions should consider including HL mechanisms in interventions designed to improve communication.

## Introduction

As reported already, COVID-19 is a disease caused by the strain SARS-CoV-2, which appeared in Wuhan, China, in December 2019, being declared as a pandemic in March 2020 (1). By now, about 116,166,652 people have been infected and 2,582,528 have died as a major consequence of this disease (2). During the first quarter of the year, several countries have declared a state of emergency, adopting and urging the adoption of preventive measures to avoid a greater dispersion of the disease (1).

COVID-19 is a disease whose clinical condition is mainly characterised by asymptomatic or mild respiratory symptoms; however, other symptoms may occur (1, 3–11). At the time of conduct of this study, no effective and/or approved antiretroviral treatment or vaccine targeting SARS-CoV-2 and the treatment is mainly symptomatic and organ support (5, 12). Thus, in this context, preventive measures such as correct handwashing, respiratory etiquette, disinfection of surfaces, social isolation and/or social distancing, and the use of masks presented themselves as preponderant measures to control this pandemic and to the individual and community protection (1, 3, 5). However, to be successful, the obligatoriness of these preventive measures requires a productive health literacy (HL) program, endowing the individuals of the ability to understand and how to follow these guidelines, and how to make decisions related to self and community health (13).

Health literacy is the ability of an individual to obtain, understand, and use the information to develop the skills to make free and informed decisions about the health of an individual and assuming an active role in preventive medicine and health policies, including the organisational and social context (14–16). Therefore, considering the actual public health problem, HL seems to have a preponderant impact through individual and community empowerment (17–19). Since there is a strong association between HL and several diseases, HL is obtaining increasing importance among the public health sector worldwide (20–23).

Health literacy is associated with three domains: primarily the functional domain, e.g., the basic skills for reading and writing health information; the interactive domain, which implies a comprehension of this information; the critical domain, which represents a more advanced stage of HL, representing the critical evaluation of health information and making informed and conscious decisions related to a self or community health (13).

Preceding, during, and after a pandemic, there are different psychological reactions arising from new necessary strategies, i.e., isolation and contact restrictions (13, 24). The consequent anxiety and, probably, fear led to a phenomenon known as “information epidemic” (infodemic)—the rapid production, spread, and amplification of information—scientifically reliable or unreliable—enhanced by an associated infodemic and its consumption (25). This phenomenon is related to higher levels of disinformation, misinformation, and malinformation, and also with two extreme attitudes: negative information bias (catastrophic thinking) and positive information bias (unrealistic optimism) (25). Moreover, the consumption of information about pandemics in Portugal was found to be associated with poor mental health indicators (25).

Therefore, COVID-19 HL can facilitate distinguishing between correct or incorrect information on COVID-19 and also empowering people to make informed and conscious decisions, and understanding and criticising the advocated political measures, being a necessary act for effective behaviour change (13, 17, 19, 25–28). Higher levels of HL usually mean higher probabilities to self-engage in health-promoting behaviours and, therefore, better health outcomes—individual and community (20, 21, 23, 29, 30).

Thus, it seemed imperative to understand and state the positive predictors of the functional COVID-19 domain of HL to establish a pattern and create HL promoting programs of COVID-19 and to act near those with lower HL.

Thereby, this study aims at understanding the predictors of HL of COVID-19—functional domain among a mainly rural district in Portugal, to promote prevention programs and provide scientific recommendations for the prevention of COVID-19/pandemics in the future.

## Materials and Methods

### Questionnaire Development

The questions included in “COVID-19’s Questionnaire” were decided on by the authors of this article. The first author selected an extensive list of important topics to cover and the other three authors independently selected the topics to be included. The final decisions were made by consensus of the authors of this article. The pilot questionnaire was applied to a group of patients to verify its comprehension and adequacy, and also the average time required to answer the questions, and a preliminary study was conducted with a smaller sample (31). The final questionnaire was applied using an online platform and divulging it through social media.

### Translation

The approved and applied version of the Questionnaire of COVID-19 is written in Portuguese. There is not any validated translation of Questionnaire COVID-19.

### Subjects and Data Collection

This cross-sectional study was performed with a sample of 864 subjects, with age older or equal to 15 years who answered to Questionnaire of COVID-19, between April 23 and June 23, 2020. We collected the demographical and epidemiological data (age, gender, education level—graduate vs. undergraduate—and risk factor(s) for COVID-19 codified by International Classification of Primary Care, version 2). Free and informed consent was obtained at the beginning of the Questionnaire of COVID-19.

This study was submitted for approval and approved by the Direction of Department of Primary Health Care of Unidade Local de Saúde do Nordeste (Ethics Committee was informed about this study but regarding pandemics of COVID-19, this Committee did not have the opportunity to assemble and adjudge this study, transferring this responsibility to the Direction of Department of Primary Health Care of Unidade Local de Saúde do Nordeste), according to the Declaration of Helsinki of the World Medical Association. The confidentiality of the data was guaranteed and was only accessible by the main investigator and the respective authors.

### Construction of the Index of Health Knowledge of COVID-19 Questionnaire (IHK-COV19)

Using the Questionnaire of COVID-19, we have constructed an IHK-COV19. For the construction of this Index, for a correct answer we have assigned two points, for an answer of an unknown concept (e.g., “I don’t know”) we have assigned 1 point, and for an incorrect answer/misconception we have assigned 0 points (because a person that states “I don’t know” can be compelled to search for information about the topic discussed while a person with an incorrect concept is someone with a higher probability of having closed boundaries in knowledge and acting according to this misconception) (32). For the questions with open answers (question 2, the symptoms of COVID-19 and question 4, the preventive measures to adopt), we adopt a system of “stated” vs. “unstated,” assigning 1 point for each symptom/preventive measure stated and 0 points for each symptom/preventive measure non-stated. The IHK-COV19 is the sum score according to the answers given by the participants.

### Statistical Analysis

All analyses were carried out using the statistical software package IBM SPSS^®^ Statistics (standard version 22.0; SPSS, Chicago, Illinois, USA) and R: a language and environment for statistical computing (version 3.6.2; R Core Team, R Foundation for Statistical Computing, Vienna, Austria). An exploratory analysis was performed to demographically characterise our sample (age, gender, education, and risk factors for COVID-19) and for the answers given for each question of our questionnaire. IHK-COV19 was taken as a continuous variable. Mixed effects models (or generalised linear mixed effects models) were used to estimate the potential predictors of IHK-COV19, regarding the four independent variables such as age, gender, education, and risk factors for COVID-19. Univariate analyses were performed to determine the relationship between each Health Knowledge Questionnaire question. Multivariate analyses were performed to determine the relationship between each IHK-COV19 with “Age,” “Gender,” “Education,” and “Risk Factor” factors. Equation of the applied model:


$$\begin{array}{l} IHK-COV19_{i}=\beta_{0}+\beta_{1}Age_{i}+\beta_{2}Gender_{i}+\beta_{3}Education_{i}\\ +\beta_{4}RiskFactor_{it}+u_{i}+\varepsilon_{i} \end{array}$$

where _*i*_ = *1, …*, 864, *ε*_*i*_ is the random error such that *ε*_*i*_~ *N* (0, σ^2^), general correlation matrix, with no additional structure; *u*_1*i*_ random effect and *u*_1*i*_~ *N* (0, d^2^).

The level of significance for all statistical tests was set at a *p*-value <5%, with a 95% CI. The confidentiality of the data was guaranteed, only accessible by the main investigator and the respective authors.

## Results

**Figure 1:**
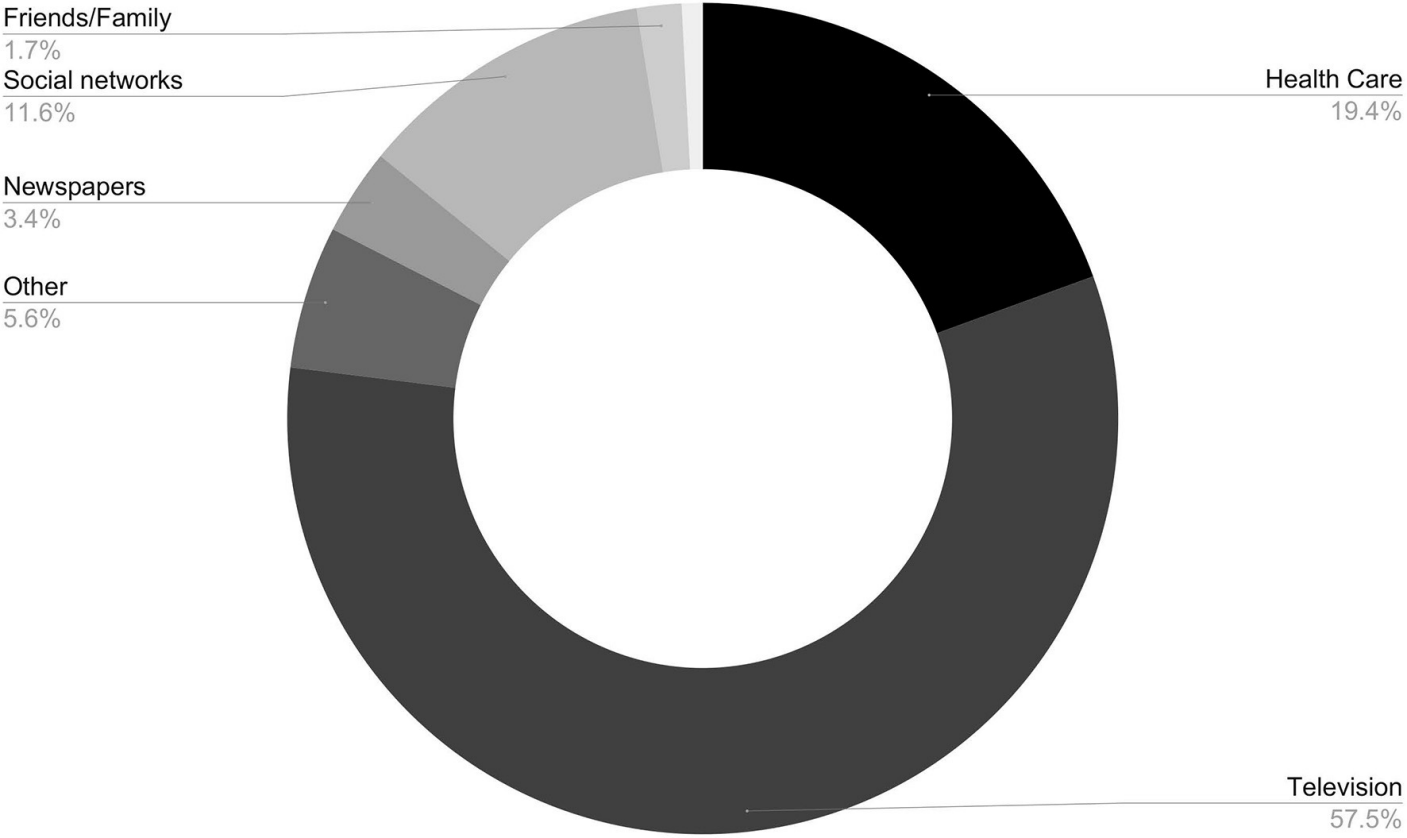
Major information sources of participants residing in the district of Bragança, Portugal, from April 23 to June 23, 2020.

The total number of participants was 864. The average age was 44.33 years old (SD = 16.07 years) and about 71.53% of the responders were women. Also, more than 50% of the individuals were graduates and 69.21% do not present a risk factor for COVID-19 (Table 1). The frequencies for each question that contributed to the IHK-COV19 are shown in Table 2. The major information sources of the participants are shown in Figure 1.

**Table 1:** Covariables of adult participants (*n* = 864) residing in the district of Bragança, Portugal, April 23 to June 23, 2020.

**Variables**	**Level/units**	**Absolute frequency** **(***n***)**	**Relative frequency (%)**
Gender	Female	620	71.76
	Male	244	28.24
Education	Undergraduate	392	45.37
	Graduate	472	54.63
Risk factor	Non-risk factor	598	69.21
	Risk factor	266	30.79
Age, years (15; 100)		Mean	Standard deviation (s.d.)
		44.33	16.07

**Table 2:** Health Literacy Questionnaire [Index of Health Knowledge of COVID-19 Questionnaire (IHK-COV19)] scores of adult participants residing in the district of Bragança, Portugal, from April 23 to June 23, 2020.

**Question**	**Levels**	**Absolute frequency** **(***n***)**	**Relative frequency (%)**
1. Do you know which are the symptoms of COVID-19?	No	31	3.59
	Yes	833	96.41
2. What are they? “Fever”	Not stated	122	14.12
	Stated	742	85.88
2. What are they? “Cough”	Not stated	178	20.60
	Stated	686	79.4
2. What are they? “Dyspnea”	Not stated	176	20.37
	Stated	688	81.71
2. What are they? “Others”	Not stated	342	39.58
	Stated	522	60.42
3. Does COVID-19 have a cure?	No	177	20.49
	I don’t know	293	33.91
	Yes	394	45.6
4. Which are the preventive measures to adopt face to the COVID-19 pandemic? “Social isolation”	Not stated	409	47.34
	Stated	455	52.66
4. Which are the preventive measures to adopt face to the COVID-19 pandemic? “Handwashing”	Not stated	357	41.32
	Stated	507	58.68
4. Which are the preventive measures to adopt face to the COVID-19 pandemic? “Respiratory Etiquette”	Not stated	767	88.77
	Stated	97	11.23
4. Which are the preventive measures to adopt face to the COVID-19 pandemic? “Other”	Not stated	220	25.46
	Stated	644	74.54
5. What are you supposed to do in case you have the symptoms of COVID-19?	Incorrect	90	33.91
	I don’t know	12	20.49
	Correct	762	45.6
6. Which is the number of SNS 24?	Incorrect	246	28.47
	I don’t know	91	10.53
	Correct	527	61
7. In social isolation, can you receive or visit family or friends at home?	Yes	36	4.17
	I don’t know	13	1.05
	No	815	94.33
8. Does COVID-19 only affect the elderly?	Yes	28	3.24
	I don’t know	4	0.46
	No	832	96.3
9. Does the use of gloves always prevent the infection by the new Coronavirus?	Yes	117	13.54
	I don’t know	51	5.9
	No	696	80.56
10. Does the use of masks always prevent the infection by the new Coronavirus?	Yes	201	23.26
	I don’t know	53	6.13
	No	610	70.6
11. Can children get sick with COVID-19?	No	829	95.95
	I don’t know	20	2.31
	Yes	15	1.74
12. Can children transmit this disease?	No	803	92.94
	I don’t know	48	5.56
	Yes	13	1.5

Based on the formulation of the models, the complete models were estimated: Estimates of fixed effects, SEs, test statistic values, and proof values. The Maximum Restricted Likelihood Method (REML) was used to adjust the different models. The analysis of residues is a useful tool for verifying the assumptions of the models regarding the AIC concentration variable. In the adjusted values vs. standardised residuals, adjusted values vs. observed values, and the QQ plot graph, we observed a pattern of homoscedasticity and few outliers. The graphical representation of the observed values and adjusted values shows that it is linearly available, and it is noticeable that there are few outliers. The representation of theoretical and empirical quantiles suggests that the residuals follow approximately a normal distribution.

Regarding each question individually, the covariables “Gender,” “Education,” and “Risk Factor” are significantly associated with the outcome. The IHK-COV19 is significantly associated with the covariable “Education,” “Age,” and “Risk factor” (Table 3); the graduated participants have the highest level of IHK-COV19 as compared with the undergraduate participants. Indeed, if an individual belongs to the Graduate Group, he has an increase of 8.2315071 in the IHK-COV19 than an ungraded one. Regarding the covariable, “Gender,” a male individual has an increase of 0.6675812 in having a higher IHK-COV19 compared with a female individual. If the individual belongs to the risk factor group, he has a decrease of 2.8946244 in IHK-COV19 than an individual belonging to the non-risk factor group (Table 3). Regarding the variable “Age,” mean age 44 years, for an increase of 1 year of age, the subject has an increase of 0.3257666 of a higher IHK-COV19.

**Table 3:** Associations between health literacy (HL), sociodemographic factors, identified in univariate and multivariate analyses, among adult participants residing in the Bragança district, Portugal, from April 23 to June 23, 2020.

	**Value**	**Std. error**	***p*** **-value**
**Fix effects**			
Intercept	21.478	0.348	
Age	−0.041	0.007	<0.01
Gender	−0.689	0.216	0.182
Education	1.369	0.206	<0.01
Risk factor	−1.089	0.242	<0.01
**Random effects**			
*d*	0.994		
AIC	4274.192		
BIC	4307.483		
logLik	−2130.096		
*R^2^*	0.902		
*R${}_{adj}^{2}$*	0.903		

## Discussion

According to the WHO, “health literacy implies the achievement of a level of knowledge, personal skills and confidence to take action to improve personal and community health by changing personal lifestyles and living conditions. […] By improving people’s access to health information, and their capacity to use it effectively, health literacy is critical to empowerment” (14). Indeed, HL is a multilayered concept, and the ability to obtain, understand and use the information to make free and informed decisions about the health of an individual (31, 33). Besides, HL plays a major role in community empowerment: a higher degree of HL means an individual proactive in preventive individual and community medicine and in effectively advocating political leaders and policymakers (15, 34). This study uses an HL, functional domain assessment tool, with a multidimensional character, to explore potential positive or negative predictors of health knowledge regarding COVID-19. The IHK-COV19 is an index based on a questionnaire that allowed us to explore the additional HL needs, and the strengths were not assessed in previous studies on behaviour in a pandemic situation, by the time of the design of this study.

Our results demonstrate that the variable “Education” is a strong positive predictor for the IHK-COV19. This result indicates that individuals with lower degrees of education might have a greater need for improvement in HL.

In the literature, several studies advocate that a higher degree of education is a predictor of a higher level of HL. Indeed, Sørensen et al. in their work on European Health Literacy Survey (HLS-EU) reported low-level education as a predictor of low HL in its different strands (14, 22, 35). Furthermore, countless scientific articles report a low degree of education as a predictor of low HL and, therefore, a predictor of poor control of a chronic disease such as asthma, diabetes, and heart failure (34–38). Kyung Lee et al. advocate that this education-HL correlation may be a consequence of social factors: lower educational levels are usually correlated to lower socioeconomic status, which may influence an increased risk of cognitive impairment due to poor nutritional intake, less access to healthcare services, social activity, interpersonal and community communication, with major social isolation (20).

Globally, men, older individuals, graduates, and individuals from the risk factor group have a better chance of having a higher IHK-COV19. Some of these findings are controversial with the literature and even with our previous study. This inconsistency might be a result of different cultural backgrounds such as educational inequalities and a potential divulgation bias (20, 35, 38–42). Another interesting study by van der Heide et al. endorses that stress or lack of concentration and motivation may affect the ability of people to understand and use health information (38). The fact that younger individuals belong to the proletariat may justify this difference regarding age.

Interestingly, Jin Lee et al., in their study, advocate that the higher the age, the more important is the role of educational level in acquiring HL (36). This finding may support the results in our study; the individuals that answered our questionnaire present a high median age, which may reinforce the role of education in the health knowledge outcome.

Even though there seems to be a strong positive correlation between the level of education and health knowledge, there are studies that plead the theory that educational level can be overcome by functional HL, because it is a process by which an individual acquires current health-related numeracy and literacy skills instead of unspecific skills obtained by formal education (38, 40). To prosecute this main objective, there are already some highlights in a recent scientific investigation. Indeed, we believe that this study reveals the importance of education and training, associating skills, and critical thinking (43). Besides establishing didactic education of healthcare professionals, it would be useful to adapt health information in a way it can be more easily accessed and understood, using some new methodologies to target the population, such as the use of short message service (33, 38, 43–45). Furthermore, the creation of training programs, along with a cohesive interrelation between healthcare professionals/social or health settings and population in general, would be a fruitful measure (34, 46, 47).

However, there are some limitations to this study. First, we did not categorise the “Education” in its different categories, i.e., <4 years of schooling; 4 years of schooling; 6 years of schooling; 9 years of schooling; 12 years of schooling; degree of bachelor; graduation; degree of master; doctorate, which may infer a bias. Thus, further research is needed to understand the outcome of HL among different levels of “Education” and to understand how to approach HL, improving it and reducing education-related disparities in health. Besides, another major limitation of the study is the fact that the study was conducted using a convenience sample from a specific district, therefore, it is not possible to extrapolate the results to the general population of Portugal.

Furthermore, in this study, we focused on education-related disparities in HL, but we did not assess the socioeconomic status of our participants or other social factors. Indeed, lower educational levels and socioeconomic status may influence a lower HL by an increased risk of cognitive impairment because of the poor nutritional intake and a higher predisposition to social isolation (20, 48). Finally, even though we adopted the general precautions, avoided communication errors, used simple language, and conducted preliminary pilot testing, it is not evident that these measures translate the understanding of all individuals (30).

## Conclusions

In this study, we focused on understanding whether age, gender, education, and risk factors for COVID-19 have an important role in health knowledge regarding this pandemic. The results found that the level of “Education” is a strong positive predictor of health knowledge outcome; the higher the level of “Education,” the higher the health knowledge regarding COVID-19.

Future studies should consider the inclusion of the variable “Education” as it may have a positive impact in the functional domain of HL of several areas.

## Data Availability Statement

The raw data supporting the conclusions of this article will be made available by the authors, without undue reservation.

## Ethics Statement

The studies involving human participants were reviewed and approved by Direção dos Cuidados de Saúde Primários da Unidade Local de Saúde do Nordeste. Written informed consent to participate in this study was provided by the participants’ legal guardian/next of kin.

## Author Contributions

JG wrote the first draught of the manuscript. CS, BA, and PM wrote sections of the article. All authors contributed to conception and design of the study, data collection and organisation of the database, statistical analysis and interpretation, manuscript revision, read, and approved the submitted version.

## Funding

This work has been partially funded by National funds, through the Foundation for Science and Technology (FCT) - project UIDB/50026/2020 and UIDP/50026/2020; and by the projects NORTE-01-0145-FEDER-000013 and NORTE-01-0145-FEDER-000023, supported by Norte Portugal Regional Operational Programme (NORTE 2020), under the PORTUGAL 2020 Partnership Agreement, through the European Regional Development Fund (ERDF).

## Conflict of Interest

PM has received in the past 3 years grants, CME-related honoraria, or consulting fees from Angelini, AstraZeneca, Bial Foundation, Biogen, DGS-Portugal, FCT, Janssen-Cilag, Gulbenkian Foundation, Lundbeck, Springer Healthcare, Tecnimede and 2CA-Braga. The remaining authors declare that the research was conducted in the absence of any commercial or financial relationships that could be construed as a potential conflict of interest.

## Publisher's Note

All claims expressed in this article are solely those of the authors and do not necessarily represent those of their affiliated organizations, or those of the publisher, the editors and the reviewers. Any product that may be evaluated in this article, or claim that may be made by its manufacturer, is not guaranteed or endorsed by the publisher.
