# Supplementary material for: Education as a Predictor Factor for Knowledge of COVID-19 in Portugal
Source: Front Public Health. 2021 Sep 29;9:680726. doi: 10.3389/fpubh.2021.680726 (PMC8516069; doi:10.3389/fpubh.2021.680726)
Supplement: Supplementary file 1 [file Data_Sheet_1.PDF]

COVID-19'S QUESTIONNAIRE

EPIDEMIOLOGIC DATA

1. County of residence (closed answer - the individuals only could select one): [PT: Distrito no qual reside]
    a. Alfândega da Fé
    b. Bragança
    c. Carrazeda de Ansiães
    d. Freixo de Espada à Cinta
    e. Macedo de Cavaleiros
    f. Miranda do Douro
    g. Mirandela
    h. Mogadouro
    i. Torre de Moncorvo
    j. Vila Flor
    k. Vimioso
    l. Vinhais

2. Gender (closed answer - the individuals only could select one): [PT: Sexo]
    a. Female [PT: feminino]
    b. Male [PT: masculino]

3. Age (short numeric open answer - the individuals only could answer with a number) [PT: Idade]

4. Schooling (closed answer - the individuals only could select one): [PT: escolaridade]
    a. <4 years of schooling [PT: 4 anos de escolaridade]
    b. 4 years of schooling [PT: 4 anos de escolaridade]
    c. 6 years of schooling [PT: 6 anos de escolaridade]
    d. 9 years of schooling [PT: 9 anos de escolaridade]
    e. 12 years of schooling [PT: 12 anos de escolaridade]
    f. Bachelor's degree [PT: Bacharelato]
    g. Graduation [PT: Licenciatura]
    h. Master's Degree [PT: Mestrado]
    i. Doctorate [PT: Doutoramento]

5. Please select the diseases that apply to you - in case you don't have any, please select "None". In case you have another disease that it isn't specified please select "Other" and state which disease you have. (closed answer - the individuals only could select one - in case the individual selected "Other", an open form field appeared and the individual could specify the disease [PT: Selecione as patologias que se aplicam a si - em caso de não ter nenhuma selecione "nenhuma". Em caso de ter outra doença que não esteja especificada selecione "Other" e especifique.]
    a. None [PT: Nenhuma]
    b. Asthma [PT: Asma]
    c. Arterial Hypertension [PT: Hipertensão Arterial]
    d. Diabetes Mellitus [PT: Diabetes]
    e. Cardiac Insufficiency [PT: Insuficiência cardíaca]
    f. Chronic Liver Disease [PT: Doença hepática crónica]
    g. Chronic Renal Disease [PT: Doença Renal Crónica]

h. Active malignant neoplasm e.g. malignant cancer [PT: Neoplasia maligna ativa i.e., cancro maligno]
i. Immunosuppression State [PT: Estado de imunossupressão]
j. Chronic Obstructive Pulmonary Disease [PT: Doença Pulmonar Obstrutiva Crónica]
k. Other [PT: Outra]

QUESTION 1: “Do you know which are the symptoms of COVID-19, the disease caused by the new Coronavírus?” | A: “Yes” OR “No”.

[P: “Sabe quais são os sintomas da COVID-19, a doença provocada pelo novo Coronavírus?” | R: “Sim” OU “Não”]

QUESTION 2: “What are they?” | A: “Fever”; “Cough”; “Dyspnea”; “Other” - Open answer [P: “Quais são?” | R: “Febre”, “Tosse”, “Dispneia”, “Outro”]

QUESTION 3: “Does COVID-19 have a cure?” | A: “Yes” OR “I don’t know” OR “No” [P: “A COVID-19 tem cura?” | R: “Sim” OU “Não sei” OU “Não”]

QUESTION 4: “Which are the preventive measures to adopt face to the COVID-19’s pandemic, i.e., to face to the actual health problem?” | A: “Social isolation”, “Handwashing”, “Respiratory Etiquette”, “Other” - open answer

[P: “Quais as medidas preventivas a adoptar perante esta pandemia, isto é, perante o problema de saúde atual?” | R: “Isolamento social”, “Higienização das mãos”, “Etiqueta Respiratória” e “Outra”]

QUESTION 5: What is supposed to do in case you have the symptoms of COVID-19 | A: “Stay at home and call SNS 24” OR “I don’t know”.

[P: “O que é que é suposto fazer caso tenha sintomas de COVID-19?” | R: “Ficar em casa e ligar para o SNS 24” OU “Não sei”]

QUESTION 6: Which is the number of SNS 24?” | A: “808 24 24 24” OR “I don’t know”

[P: “Qual o número da linha de Saúde 24?” | R: “808 24 24 24” OU “Não sei”]

QUESTION 7: “In social isolation, can you receive or visit family or friends at home?” | A: “Yes” OR “I don’t know” OR “No”

[P: “Em isolamento profilático, pode visitar ou receber familiares ou amigos em casa?” | R: “Sim” OU “Não sei” OU “Não”]

QUESTION 8: “Does COVID-19 only affect the elderly, i.e., older people?” | A: “Yes” OR “I don’t know” OR “No”

[P: “A COVID-19 só afeta os idosos, isto é, as pessoas com mais idade?” | R: “Sim” OU “Não sei” OU “Não”]

QUESTION 9: “Does the use of gloves always prevent the infection by the new Coronavírus?” | A: “Yes” OR “I don’t know” OR “No”

[P: “O uso de luvas previne sempre a infeção pelo novo Coronavírus?” | R: “Sim” OU “Não sei” OU “Não”]

QUESTION 10: “Does the use of masks always prevent the infection by the new Coronavírus?” | A: “Yes” OR “I don’t know” OR “No”

[P: “O uso de máscara previne sempre a infeção pelo novo Coronavírus?” | R: “Sim” OU “Não sei” OU “Não”]

QUESTION 11: “Can children get sick with COVID-19?” | A: “Yes” OR “I don’t know” OR “No”

[P: “As crianças podem ficar doentes com COVID-19?” | R: “Sim” OU “Não sei” OU “Não”]

QUESTION 12: “Can children transmit this disease?” | A: “Yes” OR “I don’t know” OR “No”

[“As crianças podem transmitir esta doença?” | R: “Sim” OU “Não sei” OU “Não]

QUESTION 13: “Which is your main source of information about COVID-19?” | A: “ Health professionals” OR “Friends” OR “Family” OR “Radio” OR “Social networks” OR “Newspapers” OR “Television” OR “Other” - open answer

[P: “Qual a sua principal fonte de informação acerca da COVID-19?” | R: “Profissionais de saúde” OU “Amigos” OU “Família” OU “Rádio” OU “Redes sociais” OU “Jornais” OU “Televisão” OU “Outro”]
